# Comparison of the In Vitro Susceptibility of Ceftolozane-Tazobactam With the Cumulative Susceptibility Rates of Standard Antibiotic Combinations When Tested Against *Pseudomonas aeruginosa* From ICU Patients With Bloodstream Infections or Pneumonia

**DOI:** 10.1093/ofid/ofz240

**Published:** 2019-05-20

**Authors:** Dee Shortridge, Michael A Pfaller, S J Ryan Arends, Janet Raddatz, Daryl D DePestel, Robert K Flamm

**Affiliations:** 1 JMI Laboratories, North Liberty, Iowa; 2 University of Iowa College of Medicine, Iowa City, Iowa; 3 Merck & Co, Inc., Kenilworth, New Jersey

**Keywords:** antibiogram, Ceftolozane/tazobactam, ICU, *P. aeruginosa*

## Abstract

**Background:**

*Pseudomonas aeruginosa* remains an important cause of hospital-acquired infections in the United States and is frequently multidrug-resistant (MDR). The Infectious Diseases Society of America guidelines recommend empiric combination therapy that includes an antipseudomonal β-lactam with an aminoglycoside or fluoroquinolone likely to cover ≥95% of *P. aeruginosa* infections in seriously ill patients at risk of having an MDR pathogen. Ceftolozane is an antipseudomonal cephalosporin, combined with the β-lactamase inhibitor tazobactam. Ceftolozane-tazobactam is approved for treatment of complicated urinary tract infections and complicated intra-abdominal infections. A phase 3 clinical trial for the treatment of hospital-acquired pneumonia including ventilator-associated pneumoniae was recently completed. We compared the in vitro susceptibility rate of ceftolozane-tazobactam with the cumulative susceptibility rates of antibiotic combinations commonly used against *P. aeruginosa.*

**Methods:**

Isolates were collected from intensive care unit patients hospitalized in 32 US hospitals from 2011 to 2017. The susceptibilities of 1543 *P. aeruginosa* isolates from bloodstream infections (198 isolates, 12.8%) or pneumonia (1345 isolates, 87.2%) were determined for ceftolozane-tazobactam and comparators.

**Results:**

The most active antimicrobials were colistin (99.4% susceptible), amikacin (98.1% susceptible), and ceftolozane-tazobactam (96.5% susceptible). The susceptibilities to other antipseudomonal β-lactams and fluoroquinolones were <84%. A cumulative susceptibility of ≥95% was reached for cefepime, ceftazidime, meropenem, and piperacillin-tazobactam only in combination with amikacin due to the lower susceptibilities of gentamicin, ciprofloxacin, and levofloxacin. Monotherapies that exceeded 95% were ceftolozane-tazobactam, amikacin, and colistin.

**Conclusions:**

Ceftolozane-tazobactam monotherapy is likely to be active against more isolates than a combination of another β-lactam and a fluoroquinolone or gentamicin for serious *P. aeruginosa* infections.

*Pseudomonas aeruginosa* remains an important cause of hospital-acquired infections in the United States and is frequently resistant to 3 or more drug classes (multidrug-resistant [MDR]) [1]. MDR *P. aeruginosa* is common in infections caused by *P. aeruginosa* including bloodstream infections (14.7%) and pneumonia (22.0%), which makes treating serious *P. aeruginosa* infections challenging [2]. Furthermore, delaying appropriate antimicrobial therapy has been associated with increased morbidity and mortality. Patients with MDR *P. aeruginosa* have a higher 30-day mortality than patients with non-MDR *P. aeruginosa* [3]. As such, the Infectious Diseases Society of America (IDSA) guidelines for management of adult patients with hospital-acquired and ventilator-associated pneumonia (HAP/VAP) recommend empiric combination therapy that includes an antipseudomonal β-lactam with an aminoglycoside or fluoroquinolone likely to cover ≥95% of pathogens for *P. aeruginosa* infections if the patient has risk factors for having an infection caused by an MDR isolate or if the patient is at risk of death [4]. However, due to increasing resistance to fluoroquinolones and traditional β-lactams in *P. aeruginosa*, not all antimicrobial combinations may reach susceptibility of 95%. Although aminoglycoside resistance varies by agent, with amikacin being the most active, monotherapy is not recommended for *P. aeruginosa* infections due to poor treatment outcomes [4, 5].

Ceftolozane is an antipseudomonal cephalosporin combined with the established β-lactamase inhibitor tazobactam. Ceftolozane-tazobactam is stable against the most common pseudomonal resistance mechanisms driven by mutation [6]. It has demonstrated in vitro activity against *P. aeruginosa*, including MDR isolates nonsusceptible to other β-lactam agents [7, 8]. Ceftolozane-tazobactam has been shown to be safe and effective in treating complicated urinary tract infections and complicated intra-abdominal infections (in combination with metronidazole) caused by gram-negative organisms, including *P. aeruginosa* [9]. A phase 3 clinical trial for the treatment of HAP, including VAP in intensive care unit (ICU) patients, that compared ceftolozane-tazobactam with meropenem was recently completed (NCT02070757).

In this study, we describe the activity of ceftolozane-tazobactam and comparators against *P. aeruginosa* isolates collected from ICU patients hospitalized with bloodstream infection or pneumonia in 32 US hospitals that participated in the Program to Assess Ceftolozane-Tazobactam Susceptibility (PACTS), which is part of the SENTRY Antimicrobial Surveillance Program, from 2011 to 2017. Isolates were tested against β-lactams, fluoroquinolones, and aminoglycosides, the main drug classes recommended to treat *P. aeruginosa* infections, and colistin, which is used for infections caused by MDR isolates. The purpose of this study was to compare the susceptibility of *P. aeruginosa* to ceftolozane-tazobactam with the cumulative susceptibility of antimicrobials commonly used as combination therapies for seriously ill patients.

## METHODS

A total of 1543 nonduplicate *P. aeruginosa* isolates were collected prospectively from ICUs in 32 US medical centers from 2011 to 2017 as part of the SENTRY Antimicrobial Resistance Surveillance Program. Participating centers submitted clinical bacterial isolates (1 isolate per patient per infection episode) that were consecutively collected by infection type according to a common protocol, which has been previously described [10]. The common SENTRY Program protocol established the number of isolates for the target infection types and the time period during which the isolates should be collected. Each institution contributed approximately 50 consecutive isolates per target infection type. Only isolates determined to be significant by local criteria as the reported probable cause were used. Isolates included in this study were from ICU patients with bloodstream infections (198 isolates) or pneumonia (1345 isolates). Isolates were identified at each medical center using the laboratory’s standard methods and confirmed by the central laboratory (JMI Laboratories, North Liberty, IA) using a matrix-assisted laser desorption ionization time-of-flight technology mass spectrometer (Bruker, Billerica, MS) or other methods, as needed.

Minimum inhibitory concentrations (MICs) for all antibiotics were determined by the central laboratory using broth microdilution according to Clinical and Laboratory Standards Institute (CLSI) standards [11]. All ceftolozane-tazobactam and piperacillin-tazobactam MIC testing used a fixed tazobactam concentration of 4 mg/L. Quality control and interpretation of results were performed according to CLSI M100 [12]. All MIC results for ATCC quality control strains were within published ranges.

*Pseudomonas aeruginosa* isolates were considered nonsusceptible to cefepime, ceftazidime, meropenem, piperacillin-tazobactam, amikacin, gentamicin, ciprofloxacin, or levofloxacin if their MIC value was greater than the current CLSI susceptible breakpoint [12]. Isolates were called β-lactam-nonsusceptible if they were nonsusceptible to cefepime, ceftazidime, meropenem, and piperacillin-tazobactam.

Cumulative susceptibilities to common antimicrobial combinations were calculated as the number of isolates susceptible to at least 1 of the 2 antimicrobials in the combination as described in CLSI M39 [13]. Therefore, only isolates nonsusceptible to both of the antimicrobials in the combination were excluded. This does not take into account any antagonism or synergy that may be present but does reflect the increased coverage of 2 drugs over each drug alone. The antimicrobial combinations examined were antipseudomonal β-lactams with either a fluoroquinolone, colistin, or aminoglycoside, as suggested in the IDSA HAP/VAP guidelines [4].

## RESULTS

### Susceptibility to Single Antibiotics

The susceptibilities to ceftolozane-tazobactam and comparators of the 1543 *P. aeruginosa* isolates collected from ICU patients with either bloodstream infections (198 isolates, 12.8%) or pneumonia (1345 isolates, 87.2%) are shown in Table 1. The most active antimicrobials were colistin (99.4% susceptible), amikacin (98.1% susceptible), and ceftolozane-tazobactam (96.5% susceptible). The susceptibilities for the other β-lactams tested in this study were 76.3% for meropenem, 77.1% for piperacillin-tazobactam, 83.8% for cefepime, and 82.0% for ceftazidime. Susceptibility to ciprofloxacin and levofloxacin was 73.9% and 66.0%, respectively, using the recently updated CLSI breakpoints [12]. The susceptibility of ceftolozane-tazobactam tested against bloodstream infection isolates (94.4%) was similar to the susceptibility of pneumonia isolates (96.8%). Similar susceptibility rates between the 2 infection types were also observed for the other comparators (data not shown).

**Table 1.** Activity of Ceftolozane-Tazobactam and Comparator Antimicrobial Agents When Tested Against 1543 *Pseudomonas aeruginosa* Isolates

Antimicrobial Agent	MIC_50_, mg/L	MIC_90_, mg/L	MIC Range, mg/L	CLSI^a^		
				%S	%I	%R
Ceftolozane-tazobactam	0.5	2	0.03–>32	96.5	2.1	1.4
Amikacin	4	8	≤0.25–>32	98.1	0.9	1.0
Aztreonam	8	>16	≤0.12–>16	66.5	12.3	21.3
Cefepime	4	16	≤0.5–>16	83.8	9.2	7.0
Ceftazidime	2	32	≤0.25–>32	82.0	4.9	13.2
Ciprofloxacin	0.12	>4	≤0.03–>4	73.9	5.8	20.3
Colistin	1	2	≤0.5–>4	99.4		0.6
Gentamicin	2	8	≤1–>8	86.9	4.0	9.1
Levofloxacin	0.5	>4	≤0.12–>4	66.0	10.4	23.7
Meropenem	0.5	8	≤0.06–>8	76.3	8.2	15.5
Piperacillin-tazobactam	8	>64	≤0.5–>64	77.1	10.4	12.6

Abbreviations: CLSI, Clinical and Laboratory Standards Institute; MIC, minimum inhibitory concentration.

^a^Criteria as published by CLSI 2019 [12].

MIC distributions of ceftolozane-tazobactam when tested against all isolates and against isolate groups nonsusceptible to 1 or more antimicrobials are shown in Table 2. A total of 118 isolates (7.6%) were nonsusceptible to all 4 β-lactam comparators tested; 72.0% of these were susceptible to ceftolozane-tazobactam. Table 3 shows the susceptibilities of the other comparators tested against the β-lactam-nonsusceptible isolates. Only 28.0% of the β-lactam-nonsusceptible isolates were susceptible to ciprofloxacin, and 49.2% were susceptible to gentamicin. The other drugs with higher susceptibility against isolates in the β-lactam-nonsusceptible group were amikacin at 88.1% susceptible and colistin at 99.2% susceptible.

**Table 2.** Antimicrobial Activity of Ceftolozane-Tazobactam Tested Against the Main Organisms and Organism Groups of Isolates

Organism/Organism Group (No. of Isolates) Phenotype (No. of Isolates)	No. and Cumulative % of Isolates Inhibited at MIC (mg/L) of:													MIC_50_	MIC_90_
	≤0.015	0.03	0.06	0.12	0.25	0.5	1	2	4	8	16	32	>		
*Pseudomonas aeruginosa* (1543)	0	1	1	7	106	817	383	10	**72**	33	4	5	12	0.5	2
	0.0	0.1	0.1	0.6	7.5	60.4	85.2	291.8	**96.5**	98.6	98.9	99.2	100.0		
Ceftazidime-nonsusceptible (278)					0	17	60	78	**69**	33	4	5	12	2	8
					0.0	6.1	27.7	55.8	**80.6**	92.4	93.9	95.7	100.0		
Cefepime-nonsusceptible (250)					0	3	63	67	**66**	33	4	3	11	2	8
					0.0	1.2	26.4	53.2	**79.6**	92.8	94.4	95.6	100.0		
Pip/taz-nonsusceptible (354)					0	35	119	84	**69**	30	2	4	11	2	8
					0.0	9.9	43.5	67.2	**86.7**	95.2	95.8	96.9	100.0		
Meropenem-nonsusceptible (366)				0	3	98	137	47	**41**	20	4	5	11	1	8
				0.0	0.8	27.6	65.0	77.9	**89.1**	94.5	95.6	97.0	100.0		
β-lactam-nonsusceptible^a^ (118)						0	19	28	**38**	19	2	2	10	4	32
						0.0	16.1	39.8	**72.0**	88.1	89.8	91.5	100.0		
Ciprofloxacin-nonsusceptible (402)			0	1	17	108	149	53	**38**	20	3	4	9	1	4
			0.0	0.2	4.5	31.3	68.4	81.6	**91.0**	96.0	96.8	97.8	100.0		
Levofloxacin-nonsusceptible (525)				1	18	153	203	65	**45**	23	3	4	10	1	4
				0.2	3.6	32.8	71.4	83.8	**92.4**	96.8	97.3	98.1	100.0		
Amikacin-nonsusceptible (30)					0	3	8	9	**4**	1	1	1	3	2	32
					0.0	10.0	36.7	66.7	**80.0**	83.3	86.7	90.0	100.0		
Gentamicin-nonsusceptible (202)				0	5	39	78	33	**20**	14	2	3	8	1	8
				0.0	2.5	21.8	60.4	76.7	**86.6**	93.6	94.6	96.0	100.0		
Colistin-nonsusceptible (9)					0	4	3	1	**0**	0	1			1	N/A
					0.0	44.4	77.8	88.9	**88.9**	88.9	100.0				

CLSI ceftolozane-tazobactam-susceptible breakpoint indicated by the bold column [12].

Abbreviations: CLSI, Clinical and Laboratory Standards Institute; MIC, minimum inhibitory concentration; Pip/taz, piperacillin-tazobactam.

^a^β-lactam-nonsusceptible includes ceftazidime-nonsusceptible, cefepime-nonsusceptible, piperacillin-tazobactam-nonsusceptible, and meropenem-nonsusceptible.

**Table 3.** Activity of Ceftolozane-Tazobactam and Comparator Agents When Tested Against All Organisms and Organisms Nonsusceptible to Other β-Lactams

Organism/Organism Group (No. Tested)	MIC_50_, mg/L	MIC_90_, mg/L	MIC Range, mg/L	CLSI^a^	
Antimicrobial Agent				%S	%R
*Pseudomonas aeruginosa* (1543)					
Ceftolozane-tazobactam	0.5	2	0.03–>32	96.5	1.4
Amikacin	4	8	≤0.25–>32	98.1	1.0
Cefepime	4	16	≤0.5–>16	83.8	7.0
Ceftazidime	2	32	≤0.25–>32	82.0	13.2
Ciprofloxacin	0.12	>4	≤0.03–>4	73.9	20.3
Colistin	1	2	≤0.5–>4	99.4	0.6
Gentamicin	2	8	≤1–>8	86.9	9.1
Levofloxacin	0.5	>4	≤0.12–>4	66.0	23.7
Meropenem	0.5	8	≤0.06–>8	76.3	15.5
Piperacillin-tazobactam	8	>64	≤0.5–>64	77.1	12.6
Ceftazidime-nonsusceptible (278)					
Ceftolozane-tazobactam	2	8	0.5–>32	80.6	7.6
Amikacin	4	16	≤0.25–>32	92.4	3.6
Cefepime	16	>16	2–>16	26.6	36.3
Ceftazidime	32	>32	16–>32	0.0	73.0
Ciprofloxacin	1	>4	≤0.03–>4	47.8	45.7
Colistin	1	2	≤0.5–4	99.3	0.7
Gentamicin	2	>8	≤1–>8	68.0	25.5
Levofloxacin	2	>4	≤0.12–>4	39.2	48.6
Meropenem	4	>8	≤0.06–>8	46.8	44.2
Piperacillin-tazobactam	>64	>64	4–>64	5.8	66.2
Cefepime-nonsusceptible (250)					
Ceftolozane-tazobactam	2	8	0.5–>32	79.6	7.2
Amikacin	8	16	≤0.25–>32	92.8	3.6
Cefepime	16	>16	16–>16	0.0	43.2
Ceftazidime	32	>32	1–>32	18.4	70.4
Ciprofloxacin	2	>4	0.06–>4	34.0	54.8
Colistin	1	2	≤0.5–4	99.2	0.8
Gentamicin	4	>8	≤1–>8	60.0	30.0
Levofloxacin	>4	>4	≤0.12–>4	24.0	62.0
Meropenem	8	>8	≤0.06–>8	36.4	52.8
Piperacillin-tazobactam	>64	>64	2–>64	11.6	65.6
Piperacillin-tazobactam-nonsusceptible (354)					
Ceftolozane-tazobactam	2	8	0.5–>32	86.7	4.8
Amikacin	4	16	≤0.25–>32	94.9	2.5
Cefepime	16	>16	2–>16	37.4	27.8
Ceftazidime	32	>32	1–>32	26.0	54.8
Ciprofloxacin	1	>4	≤0.03–>4	46.0	44.1
Colistin	1	2	≤0.5–4	99.4	0.6
Gentamicin	2	>8	≤1–>8	70.1	23.2
Levofloxacin	2	>4	≤0.12–>4	32.8	50.0
Meropenem	4	>8	≤0.06–>8	45.2	45.2
Piperacillin-tazobactam	>64	>64	32–>64	0.0	54.8
Meropenem-nonsusceptible (366)					
Ceftolozane-tazobactam	1	8	0.25–>32	89.1	5.5
Amikacin	4	16	≤0.25–>32	94.3	3.0
Cefepime	8	>16	1–>16	56.6	20.8
Ceftazidime	8	>32	1–>32	59.6	30.9
Ciprofloxacin	2	>4	≤0.03–>4	46.0	44.1
Colistin	1	2	≤0.5–4	98.9	1.1
Gentamicin	4	>8	≤1–>8	62.6	28.4
Levofloxacin	4	>4	≤0.12–>4	23.2	60.4
Meropenem	8	>8	4–>8	0.0	65.3
Piperacillin-tazobactam	32	>64	2–>64	47.0	29.2
β-lactam-nonsusceptible^b^ (118)					
Ceftolozane-tazobactam	4	32	1–>32	72.0	11.9
Amikacin	8	32	≤0.25–>32	88.1	5.9
Cefepime	>16	>16	16–>16	0.0	57.6
Ceftazidime	>32	>32	16–>32	0.0	83.9
Ciprofloxacin	>4	>4	0.06–>4	19.5	72.0
Colistin	1	2	≤0.5–4	99.2	0.8
Gentamicin	8	>8	≤1–>8	49.2	39.8
Levofloxacin	>4	>4	≤0.12–>4	11.9	77.1
Meropenem	>8	>8	4–>8	0.0	84.7
Piperacillin-tazobactam	>64	>64	32–>64	0.0	79.7

Abbreviations: CLSI, Clinical and Laboratory Standards Institute; MIC, minimum inhibitory concentration.

^a^Criteria as published by CLSI 2019 [12].

^b^β-lactam-nonsusceptible includes ceftazidime-nonsusceptible, cefepime-nonsusceptible, piperacillin-tazobactam-nonsusceptible, and meropenem-nonsusceptible.

### Cumulative Susceptibility to Antibiotic Combinations

**Figure 1.**
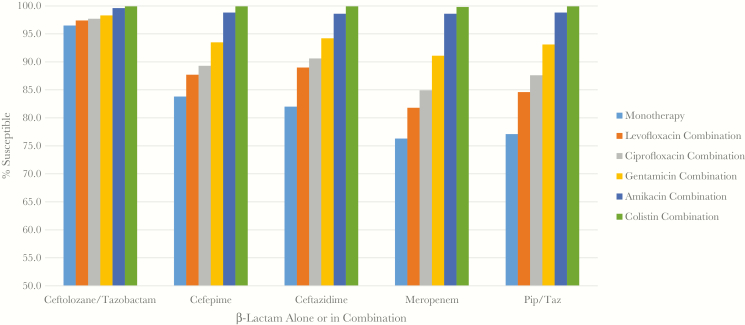
Percent of all isolates susceptible to each β-lactam alone or in combination with an agent in another class. Susceptibility based on interpretive criteria from Clinical and Laboratory Standards Institute M100 [12]. Abbreviation: Pip/Taz, piperacillin-tazobactam.

The cumulative susceptibilities of isolates to either drug in combination therapy, β-lactam with a fluoroquinolone (ciprofloxacin, levofloxacin), colistin, or aminoglycoside (gentamicin, amikacin) were calculated and are shown in Figure 1. A threshold of 95% susceptibility was used for comparison as recommended in the IDSA guidelines for management of patients with HAP/VAP [4]. Cumulative susceptibilities of ≥95% were reached for cefepime, ceftazidime, meropenem, or piperacillin-tazobactam only when combined with amikacin or colistin. Monotherapy with ceftolozane-tazobactam (96.5%), amikacin (98.1%), or colistin (99.4%) also exceeded the 95% threshold. Not surprisingly, the combinations of ceftolozane-tazobactam with either a fluoroquinolone or aminoglycoside also had >95% susceptibility, as shown in Figure 1. The combination of colistin with other β-lactams exceeded 99% susceptibility (Figure 1). Only 9 isolates were nonsusceptible to colistin, and of those, 3 isolates were also nonsusceptible to 1 or more of the β-lactams tested. When looking at the cumulative susceptibility of isolates that were nonsusceptible to other β-lactams, only the combination of ceftolozane-tazobactam with amikacin (95.8%) or colistin (100%) reached the 95% threshold.

## DISCUSSION

Empiric therapy with 2 antipseudomonal antibiotics from 2 different classes is recommended by the IDSA for HAP/VAP patients at a high risk of death with the goal of selecting agents that cover >95% of pathogens based on an institution’s antibiogram [4]. Although clinical microbiology laboratories offer standard antibiograms, most do not provide cumulative antibiograms that report the pooled susceptibilities for each of the potential combinations to help guide clinicians’ selection of ideal antipseudomonal regimens, particularly for newer agents [14, 15]. Increasing antimicrobial resistance in several classes makes selecting an active antibiotic regimen challenging [6]. Similarly, the evolution of MDR *P. aeruginosa* makes selecting a combination regimen problematic [16]. Data from this large US surveillance program show that <84% of *P. aeruginosa* isolates were susceptible to traditional β-lactam agents. Considering the lower fluoroquinolone susceptibilities (66.0%–73.9% in this study), regimens combining a generic β-lactam agent with a fluoroquinolone are also unlikely to reach the 95% threshold as shown in this study and in a recent study by Goodlet et al. [16]. A limitation of this study is that ceftazidime-avibactam and tobramycin were not included as comparators. Combining an aminoglycoside with a β-lactam improves the likelihood of reaching 95%; however, only a combination with amikacin is likely to exceed 95% as gentamicin susceptibility was only 68% in our study. Although amikacin and colistin exceeded 95% susceptibility, aminoglycosides are not recommended as monotherapy for *P. aeruginosa* infections due to increased toxicity, poor lung penetration, and lower clinical response rate [4]. Colistin monotherapy and the combination of a β-lactam with colistin have >99% susceptibility, although useing colistin is not recommended for primary therapy of VAP due to lack of pharmacokinetic and efficacy studies and drug toxicity concerns [4]. The decreased susceptibility of the β-lactam-nonsusceptible isolates to ceftolozane-tazobactam and amikacin but not colistin suggests that colistin therapy may be needed in certain situations, depending on local susceptibility rates. The pros and cons of using colistin have been discussed in other studies [17, 18].

This study included a large collection of *P. aeruginosa* isolates collected from diverse regions across the United States, specifically in ICU patients with pneumonia or bloodstream infections, and utilized a central laboratory that employs standardized reference susceptibility testing methods. Although our specific findings may not be generalizable to individual institutions, they suggest that a similar antibiogram analysis may be useful to understand the susceptibilities of local isolates to better inform the selection of empiric therapy. However, a common challenge for clinical microbiology labs arises when trying to report antibiotic susceptibilities for recently approved antibiotics. Delays in regulatory clearance of susceptibility testing devices and the practice of performing reflex testing (ie, susceptibility testing newer agents only on isolates resistant to routinely tested β-lactams) can hinder the evaluation of susceptibility testing to newer agents, thereby increasing the importance of surveillance data, including those presented therein [14].

Although antibiograms can be useful in the selection of empiric therapy, they are just 1 tool that clinicians can utilize to select appropriate empiric therapy. Other factors, including patient risk factors (eg, previous antibiotic exposure, prior microbiological data) and severity of illness (eg, critically ill, type of infection), should also be taken into clinical consideration. Rapid molecular diagnostics may also play an important role in identifying the causative pathogen and resistance phenotype, resulting in more timely selection of appropriate antibiotic therapy. However, clinical data with rapid molecular diagnostics specific to *P. aeruginosa* infections are still limited [19]. It is imperative that clinicians utilize sound antimicrobial stewardship principles, which at their core strive to optimize antimicrobial selection while limiting emergence of resistance, adverse drug events, and cost, with the ultimate goal of improving patient outcomes [20]. Importantly, empiric therapy should be re-evaluated as soon as additional data are available, including but not limited to organism identification, susceptibility, molecular data, and underlying clinical considerations, with the goal of optimizing therapy (ie, drug, dose, duration) and de-escalating therapy to limit exposure to agents that can promote resistance or toxicity.

Individual institutions should consider constructing antibiogram data with the inclusion of newer agents to evaluate empiric therapy selections for serious infections, such as pneumonia or bloodstream infections caused by *P. aeruginosa* [21]. The susceptibility of *P. aeruginosa* to ceftolozane-tazobactam, including isolates resistant to other β-lactams, may make it a useful empiric therapy in ICU patients when there is a high likelihood that MDR *P. aeruginosa* is the causative pathogen.
